# Disparities in the Use of Single-fraction Stereotactic Radiosurgery for the Treatment of Brain Metastases From Non-small Cell Lung Cancer

**DOI:** 10.7759/cureus.4031

**Published:** 2019-02-07

**Authors:** Ankit Modh, Abhishek Doshi, Charlotte Burmeister, Mohamed A Elshaikh, Ian Lee, Mira Shah

**Affiliations:** 1 Radiation Oncology, Henry Ford Hospital, Detroit, USA; 2 Radiation Oncology, Cornell University, Ithaca, USA; 3 Epidemiology and Public Health, Henry Ford Hospital, Detroit, USA; 4 Neurosurgery, Henry Ford Hospital, Detroit, USA

**Keywords:** brain metastasis, radiation, utilization, radiosurgery

## Abstract

Purpose: Radiation treatment patterns in patients with brain metastases from non-small cell lung cancer (NSCLC) have not been well elucidated. The National Cancer Database (NCDB) was used to evaluate trends in the use of whole brain radiation therapy (WBRT) and stereotactic radiosurgery (SRS) for brain metastasis from NSCLC.

Methods: This NCDB study included patients > 18 years old with metastatic NSCLC treated with single-fraction SRS or WBRT between 2004 and 2014. Chi-square, t-test, and multivariable logistic regression analyses were used to identify predictors of SRS versus WBRT.

Results: Of 40,803 patients, 34,183 (83.8%) received WBRT and 6,620 (16.2%) received SRS. SRS utilization increased from 7% (157 cases) in 2004 to 37% (1,346 cases) in 2014 (p < .001). SRS was utilized more by academic than community facilities (22% versus 13%, p < .001). The strongest independent predictors of SRS included year of diagnosis in 2010-2014 versus 2004-2009 (odds ratio [OR] 2.62, 95% CI 2.46-2.79, p < .0001), metropolitan versus rural (OR 2.26, CI 1.79-2.85, p < .0001), distance from cancer-reporting facility of ≥ 30 versus < 30 miles (OR 2.36, CI 2.18-2.56, p < .0001), private insurance versus non-insured patients (OR 1.96, CI 1.68-2.29, p < .0001), and academic versus community facility (OR 1.76, CI 1.66-1.87, p < .0001).

Conclusion: SRS for NSCLC brain metastases has steadily increased in the United States; however, WBRT remains the most commonly used. Wide geographic and socioeconomic variations exist in the utilization of SRS and WBRT for this patient population.

## Introduction

Brain metastases are the most common type of intracranial tumor and affect up to 40% of all patients with cancer. More than half of these metastases come from lung cancer histologies [1-2]. Survival from lung cancer continues to improve, which could be explained by improvements in systemic therapies including chemotherapy, targeted agents and immunotherapies [3]. Despite these advancements, the primary modalities of treatment for brain metastasis remain surgery and radiation, with a large portion of these patients receiving whole brain radiation therapy (WBRT) [4].

The value of WBRT in the treatment of brain metastasis from non-small cell lung cancer (NSCLC) has recently been questioned. The Quality of Life after Treatment for Brain Metastases trial randomized patients with brain metastasis from NSCLC who were unsuitable for surgery or stereotactic radiosurgery (SRS) to WBRT or best supportive care. With no difference found in overall survival and a small difference in the quality of life, the authors concluded WBRT provided little additional clinical benefit over supportive care [5]. WBRT also has been found to have more detrimental side effects than more focally delivered radiation, such as SRS. A recent Alliance trial randomized patients with one to three brain metastases (mostly from NSCLC) to SRS with or without WBRT. The group of patients treated with SRS alone had fewer cognitive deficits but at the expense of intracranial control; both arms had similar overall survival [6]. 

Prior randomized evidence, such as the two Patchell et al. studies, give credibility to treat these patients with WBRT, which is technically easy to plan and deliver safely to almost all patients [7-8]. While Gamma Knife (Elekta Instrument AB, Stockholm, Sweden) radiosurgery was the first platform to deliver SRS starting in 1951, SRS became widely available after refinement of linear accelerator (LINAC)-based SRS systems [9]. SRS, however, requires extensive therapy and physics staff support to deliver successfully and safely. It remains unknown if these technical challenges limit access to SRS; radiation treatment patterns in these patients are not well understood. We used the National Cancer Database (NCDB) to evaluate trends in the use of WBRT and SRS in the treatment of brain metastasis from NSCLC and factors that may reveal limitations to SRS accessibility.

## Materials and methods

The NCDB is a nationwide joint project of the Commission on Cancer of the American College of Surgeons and the American Cancer Society, serving as a powerful surveillance and quality improvement mechanism for participating cancer programs. This clinical oncology outcomes database is sourced from hospital registry data that are collected in more than 1500 Commission on Cancer accredited facilities, presenting nearly 70% of all new invasive cancer diagnoses in the United States each year [10]. The data used in the study were derived from a deidentified NCDB file. The American College of Surgeons and the Commission on Cancer have not verified and are not responsible for the analytic or statistical methodology employed or the conclusions drawn from these data by the investigators. This dataset was used in our analysis and was exempt from institutional review board authorization.

The NCDB was used to identify patients > 18 years old with metastatic NSCLC who were treated with single-fraction SRS to the brain or WBRT between 2004 and 2014. Patients receiving brain radiation dose ranges of 12-24 Gy in one fraction were classified as having received SRS, and those who received 30 Gy in 10 fractions, 20 Gy in 5 fractions, or 37.5 Gy in 15 fractions were classified as having received WBRT. Patients who did not receive radiotherapy to the brain or did not receive treatment dose within these ranges were excluded.

Univariate comparisons were conducted to compare demographic, clinicopathologic, and health care system factors between those receiving SRS or WBRT. This was done using independent two-group t-tests and chi-square tests as appropriate. Multivariable logistic regression was performed to identify possible independent predictors of receiving SRS or WBRT. Multivariable models were selected by first including any predictor with a univariate p-value < .2 and then employing stepwise selection, with a p-value cutoff of .05 used to remain in the model. Cochran-Armitage tests were used to describe the trends in radiation use by decade of diagnosis. Data from the NCDB were filtered and all data analysis was performed using SAS version 9.4 (SAS Institute Inc., Cary, NC).

## Results

Of 40,803 patients identified, 34,183 (83.8%) received WBRT and 6,620 (16.2%) received SRS. Patients were most likely to be white (83%), male (52%), and aged 55-64 years (39%). The most commonly employed WBRT doses were 30 Gy in 10 fractions (24,479 cases [60%]) and 37.5 Gy in 15 fractions (9,127 cases [22%]). The common single-fraction SRS dose range included 17-21 Gy (4,022 cases [10%]). Most patients were treated at community centers (25,465 cases [63%]), which include community cancer programs, comprehensive community cancer programs, or an integrated network cancer program, as defined by the Commission on Cancer of the American College of Surgeons. A large portion (82%) of these patients were treated in a metropolitan setting (as defined by the United States Department of Agriculture Economic Research Service).

**Figure 1:**
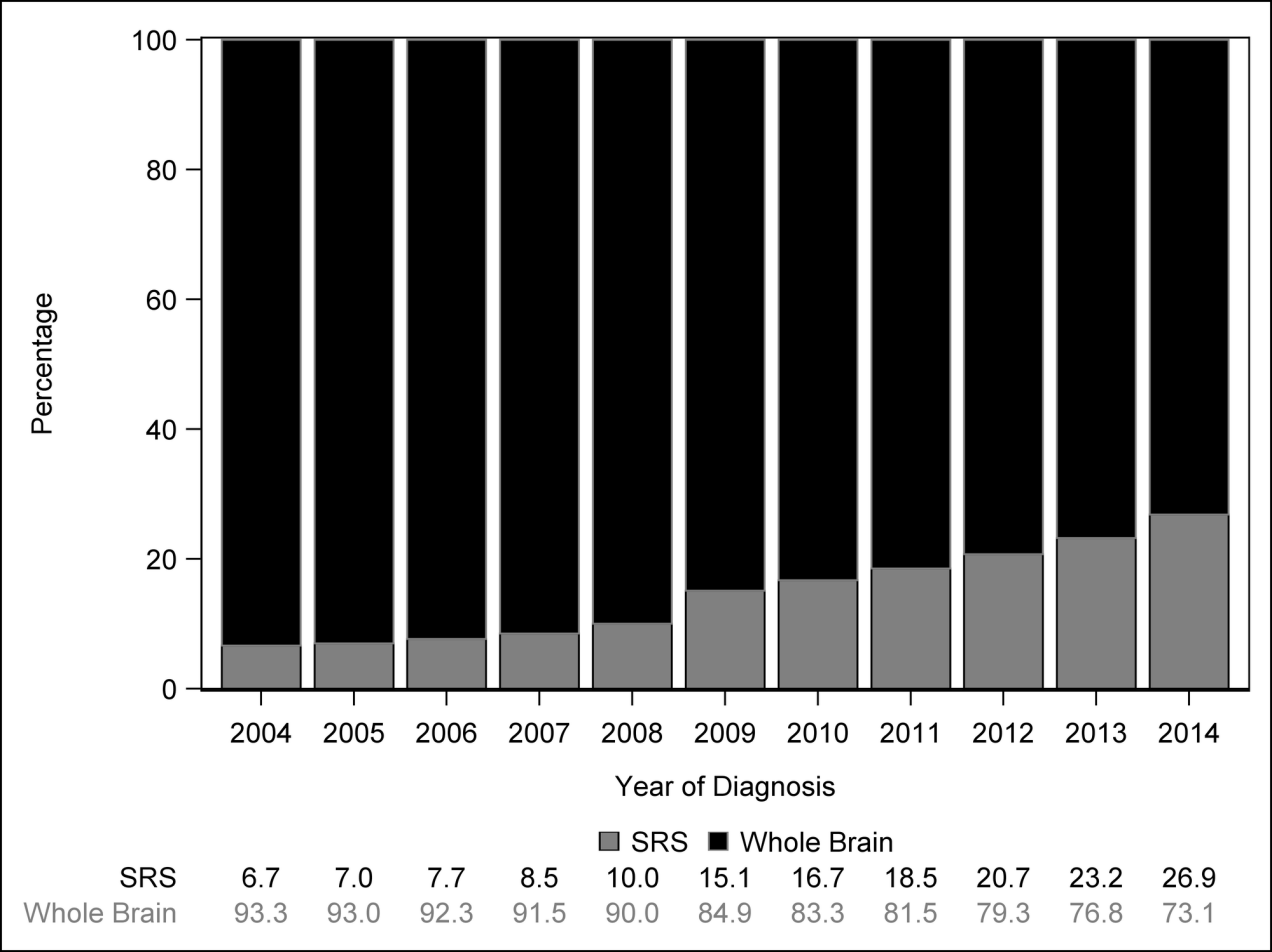
Distribution of SRS and WBRT use by year of diagnosis SRS: stereotactic surgery; WBRT: whole brain radiation therapy.

Complete patient characteristics are detailed in Table 1, which describes patient demographic, clinicopathologic, and health care system factors associated with the use of WBRT or SRS. The total number of cases treated with WBRT increased from 2,198 in 2004 to 3,662 in 2014; SRS cases increased from 157 to 1,346 over the same time period. The proportion of patients receiving SRS increased from 7% (157 cases) in 2004 to 37% (1,346 cases) in 2014 (p < .001) (Figure 1). The proportion of patients undergoing SRS delivered by LINAC versus Gamma Knife increased from 13% in 2004 to 29% in 2014 (p < .001). SRS was utilized more by academic than community facilities (22% versus 13%, p < .001).

**Table 1:** Patient demographics, clinicopathologic and health care system factors associated with WBRT or SRS WBRT: whole brain radiation therapy; SRS: stereotactic surgery.

Variable	Response	WBRT (N=34183)	SRS (N=6620)	P
Age (years)	< 55	7298 (85%)	1306 (15%)	.0180
	55-64	11242 (84%)	2110 (16%)	
	65-74	10128 (83%)	2018 (17%)	
Sex	Male	18050 (85%)	3243 (15%)	<0.0001
	Female	16133 (83%)	3377 (17%)	
Race	White	28171 (83%)	5572 (17%)	.0004
	Black/Other	5740 (85%)	986 (15%)	
	Unknown	272 (81%)	62 (19%)	
Median income quartiles		7165 (87%)	1080 (13%)	<0.0001
	$38,000-$47,999	8469 (85%)	1501 (15%)	
	$48,000-$62,999	8932 (84%)	1718 (16%)	
	$63,000 +	8818 (80%)	2228 (20%)	
Education quartiles	≥21%	6320 (87%)	945 (13%)	<0.0001
	13-20%	9579 (85%)	1681 (15%)	
	7.0-12.9%	11040 (83%)	2284 (17%)	
	<7%	6468 (80%)	1619 (20%)	
Urban	Metro	26779 (83%)	5422 (17%)	<0.0001
	Urban	5248 (86%)	859 (14%)	
	Rural	790 (89%)	95 (11%)	
Histology	Large cell	1278 (86%)	204 (14%)	<0.0001
	Squamous cell	3813 (83%)	796 (17%)	
	Adenocarcinoma	19470 (83%)	4090 (17%)	
	Other	9622 (86%)	1530 (14%)	
Charlson score	0	22908 (83%)	4610 (17%)	<0.0001
	1	7957 (85%)	1450 (15%)	
	2	3318 (86%)	560 (14%)	
Facility type	Academic	11606 (78%)	3325 (22%)	<0.0001
	Community	22239 (87%)	3226 (13%)	
Facility location	East	14089 (81%)	3224 (19%)	<0.0001
	Midwest	9908 (85%)	1716 (15%)	
	South	5186 (89%)	612 (11%)	
	West	4662 (82%)	999 (18%)	
Insurance	Not insured	2186 (91%)	216 (9%)	<0.0001
	Private insurance	12323 (83%)	2604 (17%)	
	Medicaid	3516 (87%)	538 (13%)	
	Medicare	14910 (83%)	3046 (17%)	
	Other government	618 (86%)	103 (14%)	
	Unknown	630 (85%)	113 (15%)	
Distance from reporting facility (miles)	< 10	17995 (87%)	2799 (13%)	<0.0001
	10-19	6727 (83%)	1378 (17%)	
	20-49	5992 (81%)	1390 (19%)	
	50+	2701 (74%)	963 (26%)	
Distance (miles)	< 30	27900 (85%)	4844 (15%)	<0.0001
	30+	5515 (77%)	1686 (23%)	
Year of diagnosis	2004	2198 (93%)	157 (7%)	<0.0001
	2005	2413 (93%)	182 (7%)	
	2006	2695 (92%)	225 (8%)	
	2007	2904 (91%)	271 (9%)	
	2008	3003 (90%)	334 (10%)	
	2009	3124 (85%)	557 (15%)	
	2010	3294 (83%)	662 (17%)	
	2011	3435 (81%)	782 (19%)	
	2012	3671 (79%)	960 (21%)	
	2013	3784 (77%)	1144 (23%)	
	2014	3662 (73%)	1346 (27%)	
Year of diagnosis (grouped)	2004-2009	16337 (90%)	1726 (10%)	<0.0001
	2010-2014	17846 (78%)	4894 (22%)	

On multivariable analysis, the strongest independent predictors of SRS use included year of diagnosis in 2010-2014 versus 2004–2009 (odds ratio [OR] 2.62, 95% CI 2.46-2.79, p < .0001), metropolitan versus rural location (OR 2.26, 95% CI 1.79-2.85, p < .0001), distance from cancer-reporting facility of ≥ 30 versus < 30 miles (OR 2.36, 95% CI 2.18-2.56, p < .0001), private insurance versus non-insured patients (OR 1.96, 95% CI 1.68-2.29, p < .0001), higher median income ($63,000 vs $38,000-$47,000, OR 1.12, 95% CI 1.02-1.23, p = .0172), and academic versus community facility type (OR 1.76, 95% CI 1.66-1.87, p < .0001) (Table 2).

**Table 2:** Multivariable logistic regression analysis of predictors of SRS use compared to WBRT SRS: stereotactic surgery; WBRT: whole brain radiation therapy.

Characteristic	Variable	OR (95% CI)	P
Sex	Male vs female	0.91 (0.86, 0.96)	.0012
Education quartiles	≥21% vs <7%	0.75 (0.67, 0.85)	<0.0001
	13-20% vs <7%	0.83 (0.76, 0.92)	.0003
	7-12.9% vs <7%	0.90 (0.83, 0.98)	.0123
Urban	Urban vs rural	1.42 (1.12, 1.79)	<0.0001
	Metro vs rural	2.26 (1.79, 2.85)	<0.0001
Histology	Squamous cell vs adenocarcinoma	1.13 (1.03, 1.24)	.0074
	Large cell vs adenocarcinoma	1.01 (0.86, 1.19)	.8689
	Other vs adenocarcinoma	0.93 (0.87, 1.00)	.0486
Facility	Academic vs community	1.76 (1.66, 1.87)	<0.0001
Facility location	West vs east	1.01 (0.92, 1.09)	.9151
	South vs east	0.61 (0.55, 0.67)	<0.0001
	Midwest vs east	0.75 (0.70, 0.81)	<0.0001
Insurance	Private vs not insured	1.96 (1.68, 2.29)	<0.0001
	Other government vs not insured	1.37 (1.05, 1.79)	.0223
	Medicare vs not insured	1.97 (1.69, 2.30)	<0.0001
	Medicaid vs not insured	1.36 (1.14, 1.62)	.0007
	Unknown vs not insured	1.70 (1.31, 2.20)	<0.0001
Distance (miles)	30+ vs < 30	2.36 (2.18, 2.56)	<0.0001
Median income (thousands)	<38 vs 38-47	0.93 (0.84, 1.02)	.1179
	63 vs 38-47	1.12 (1.02, 1.23)	.0172
	48-62 vs 38-47	1.00 (0.92, 1.08)	.9312
Charlson score	≥2 vs 0	0.88 (0.79, 0.97)	.0115
	1 vs 0	0.95 (0.89, 1.02)	.1303
Year	2010-2014 vs 2004-2009	2.62 (2.46, 2.79)	<0.0001

## Discussion

We present a large United States hospital-registry based study analyzing the patterns of radiation delivered for patients with brain metastasis from NSCLC. This is the first study with clinical and demographic comparisons between the utilization of WBRT and SRS [11-12]. Results reveal a socioeconomic variation between the use of either modality that should be further explored, given the increasing evidence in favor of treating brain metastasis with SRS over WBRT.

A similarly designed study by Park et al. using NCDB data revealed increased utilization over time of LINAC-based SRS for the treatment of NSCLC brain metastasis [13]. Comparable to our analysis, Gamma Knife-based radiosurgery remained the most commonly used modality, especially in academic centers. The emergence of LINAC-based systems such as the Accuray Cyberknife (Accuray Inc., Sunnyvale, California), Varian Edge or TrueBeam (Varian Medical Systems, Palo Alto, California), or the BrainLAB Novalis (BrainLAB, Munich, Germany) have made SRS more accessible and cost-effective [14]. The total number of brain metastasis cases has increased overall as has the proportion of those treated with SRS. This pattern is concordant with the abundance of studies during this era revealing the efficacy and utility of SRS.

Our analysis revealed multiple socioeconomic factors that were more common in patients treated with SRS. Insured patients (private, Medicare, or Medicaid), higher median income, or those treated in an academic facility or metropolitan setting were more likely to receive SRS over WBRT. This pattern potentially highlights an economic disparity in the delivery of SRS to patients with brain metastases. Patients from a lower socioeconomic status may present with more advanced disease requiring WBRT. This hypothesis was validated by prior studies. In their analysis of over 400,000 patients with the 10 deadliest cancers (including breast, lung, colorectal, and head and neck cancers), Walker et al. found those with less insurance coverage were more likely to present with advanced disease and receive less radiation therapy [15]. Freedman et al. reported lower odds of receiving definitive locoregional therapy and adjuvant systemic treatments for uninsured women, Medicaid enrollees, and younger Medicare beneficiaries in patients with breast cancer [16]. Patients with private insurance are more likely to receive proper cancer screening, more prompt appointments, and necessary prescription medications [17], which may lead to presenting with earlier, more treatable stages of cancer.

Additional barriers to receiving SRS include limited access to facilities that are technically capable of safely delivering radiosurgery. This is underscored by the finding that those traveling > 30 miles were more likely to receive SRS over WBRT. Travel distance has been shown to affect treatment decisions for patients with breast, colon, rectal, lung, ovarian, and prostate cancers [18-19]. Other technically challenging and resource-intensive modalities, such as brachytherapy, have also been subject to socioeconomic disparities [20]. Our study supports the message of these earlier studies and is the first, to our knowledge, to compare utilization of radiation for brain metastasis.

Our analysis has limitations. The specific patient and tumor characteristics that factored into the treatment decision of SRS or WBRT are not known. These include number and volume of metastasis, performance status, or other treatments such as immunotherapy or targeted agents. It is also unclear how many cases later received salvage therapy with further SRS or WBRT. For simplicity, our analysis included only NSCLC histologies and SRS delivered in a single fraction. The data represented here also ended in 2014. It will be interesting to see if recent randomized data and association-based guidelines such as the American Society for Radiation Oncology’s Choosing Wisely campaign have changed practice accordingly [21]. Registry data have been shown to report variable rates of actual radiation delivered [22]. Despite these limitations, our intended purpose was to elucidate radiation treatment patterns in this subset of patients and to discover factors predictive of modality utilization.

## Conclusions

The use of SRS for NSCLC brain metastases has steadily increased over time in the United States, especially in the academic setting, but WBRT remains the most common treatment modality. Wide geographic and socioeconomic variation exists in the utilization and accessibility of SRS and WBRT for this patient population.
